# Awareness and Perceptions of the Impact of Tonsillectomy on the Level of Immunity and Autoimmune Diseases Among the Adult Population in Qassim, Saudi Arabia

**DOI:** 10.7759/cureus.75219

**Published:** 2024-12-06

**Authors:** Mazyad Alenezi, Ghusun S Al Harbi, Ghaday Almutairi, Reham Almahdi, Bassam A Alharbi, Abdullah O Almutairi, Lama B Almutairi, Seba M Alraddadi

**Affiliations:** 1 Otolaryngology - Head and Neck Surgery, Qassim University, Qassim, SAU; 2 College of Medicine, Qassim University, Unaizah, SAU; 3 Unaizah College of Medicine, Qassim University, Qassim, SAU; 4 College of Medicine, Al Baha University, Al Baha, SAU; 5 College of Medicine, Qassim University, Buraidah, SAU; 6 College of Medicine, Qassim University, Unaizah, SAU

**Keywords:** autoimmune disease, immunity, knowledge, tonsil, tonsillectomy

## Abstract

Introduction

Tonsils are part of the immune system, but recurrent tonsillitis may necessitate tonsillectomy. While studies show that tonsillectomy reduces throat infections and improves quality of life, it does not significantly affect immunity or increase the risk of autoimmune diseases. Despite this, misconceptions about its impact persist. This study aimed to assess awareness of the procedure’s effects on immunity and autoimmune diseases in the adult population of the Qassim Region.

Methods

This cross-sectional study used data from a sample of 383 adults living in the Qassim Region, Saudi Arabia. An online self-administered questionnaire was distributed through social media platforms. The questionnaire included socio-demographic information (e.g., age, gender, occupation) and various questions to assess the population’s awareness and perceptions of the impact of tonsillectomy on immunity and autoimmune diseases. Participants completed the questionnaire anonymously.

Results

Of the 383 participants, 65 (17.0%) had undergone tonsillectomy. Nearly half (209; 54.6%) believed that tonsillectomy affects immunity while about one-third (141; 36.8%) thought there was a relationship between tonsillectomy and autoimmune diseases. The most common sources of information about tonsillectomy were healthcare professionals (95, 24.8%), followed by social media (91, 23.5%) and community members (88, 23%). Statistically significant associations were found between education level, occupation, monthly income, and awareness of the impact of tonsillectomy on immunity. Multivariate logistic regression analysis revealed that participants with a bachelor's degree or higher were 1.63 times more likely to be aware of the impact of tonsillectomy on immunity (AOR = 1.632; 95% CI = 1.023-2.604; p = 0.040) compared to those with lower education. However, no significant associations were found between occupation or monthly income and awareness of the impact of tonsillectomy on immunity after adjusting for these factors (p > 0.05).

Conclusion

The study found that the adult population's awareness of the impact of tonsillectomy on immunity was inadequate. Public awareness programs and social media could play a crucial role in dispelling misconceptions about the procedure's effects on immunity and autoimmune diseases. There is also a need for further educational interventions to challenge and correct public misconceptions about the relationship between tonsillectomy, immunity, and autoimmune diseases.

## Introduction

Tonsils are critical for protecting humans against oral pathogens. The two lymphoid tissues contain B cells that get activated when the tonsils come into contact with a pathogen, causing inflammation of the tonsils, commonly referred to as tonsillitis. This condition is most common among children and sometimes requires tonsillectomy, especially when it is recurrent [1].

In tonsillectomy, the tonsils are completely removed to eradicate exposure to tonsillitis. The procedure, which involves carefully dissecting the tissue around each tonsil and separating it from the muscles of the throat, can be conducted with or without adenoidectomy [2]. In the United States, tonsillectomy is one of the most commonly performed procedures, especially among children aged 15 and below, with procedures being performed annually for about 289,000 patients. Obstructive sleep-disordered breathing (OSDB) is the most cited reason for tonsillectomy, with recurrent throat infections also being a significant reason [2].

Multiple studies carried out in the USA, including a study conducted in Colorado in 2015-2016, revealed that tonsillectomy may show better results for severe throat infections. In the first two years, tonsillectomy shows less incidence than non-surgical management; in the third year, no difference appears and may show less severe and fewer episodes in the non-surgical group. Adenotonsillectomy is not recommended among patients with active infection, unmanaged systemic disease, blood disorders, or risk of velopharyngeal insufficiency.

Regarding the complications of this procedure, reports showed weight gain postoperatively, without affecting the immune function [3].

Moreover, in a study in the USA in 2011, the researcher stated that he showed increased growth hormone secretions and significant increases in IGFBP-3 and IGF-1 after tonsillectomy, which showed more in children with growth failure [4]. A cohort study done in Sweden in 2016 concluded that tonsillectomy showed a significant increase in autoimmune diseases; the study found that from 179,875 of those who went for tonsillectomy between 1997 and 2012 in Sweden, 5,357 later tested positive for autoimmune diseases [1]. Furthermore, a study conducted in Bergen, Norway, in 2014, surveyed 45 children, measuring the level of immunoglobulin M (IgM), IgG, and IgA before tonsillectomy and up to a year after; this study was able to see the results of these children's response to the live influenza vaccine and the effect of the surgical procedure on the immune response against the vaccine. The results of this study indicated that the body's humoral response to the live vaccine was not affected. This study further noted that it did not find a clear difference in the short or even long term in IgM, IgG, and IgA levels. Therefore, the study concluded that the procedure did not hurt humoral systemic immunity or specific immunity against the vaccine from this surgical procedure [5].

A recent study conducted in Belgium in 2017 showed that there is a significant reduction in the number of antibiotics and respiratory medications used and pediatrician visits during the year between, before, and after the adenotonsillectomy procedure, reflecting an improvement in infections, breathing, income, and quality of life. There is a consensus that there is no clear long-term or short-term evidence of the effect of this procedure on the body's immune processes [6]. In addition, a recent study conducted in Taiwan in 2023 on 2154 children to assess the changes in voice parameters after one and three months post-tonsillectomy by computerized acoustic analysis showed that there is a significant change in shimmer, jitter, and soft phonation index after one month, without any substantial change after three months [7]. Furthermore, a study conducted in Turkey in 2008 compared the results in the early and late phases in the long term. Its results indicated that immunity in both its types, cellular and humoral, is not affected, and when compared to healthy controls, it returns to similar levels [8]. Moreover, a 2015 review of 35 studies concluded that tonsillectomy does not affect the immune system [9]. A recent study conducted in 2023 at Abha City, Kingdom of Saudi Arabia, showed that 13% of 800 subjects 18 and above years old had a tonsillectomy, 36% believed that tonsillectomy would affect the immune function, 18% of people associate this procedure with autoimmune diseases. One-third learned this information through the community and social media [10]. Moreover, a study conducted in Saudi Arabia in 2019 of 404 children reported no notable change in the levels and components of cellular immunity before and after surgery. However, some showed a slight increase or decrease in these levels; they were insignificant and did not differ significantly from those before surgery. This result also applied to humoral immunity, as this study concluded with no negative difference in immunity after tonsillectomy [11]. In addition, a recent study conducted in China in 2024 showed that there was no adverse effect on immunity from the adenoidectomy, tonsillectomy, and adenotonsillectomy procedures, despite the presence of a large number of memory B cells in the tonsils; their removal did not affect the work of humoral immunity. One study showed an apparent decrease in IgM, IgA, and IgG levels in the study sample between the ages of four and six years. However, this decrease did not cause a clear and significant change in immune status. As for cellular immunity, studies agree that it was not negatively affected after removal [12]. As observed in clinical practice, a prevalent misconception persists within the community that tonsillectomy results in a decline in immune function. This misconception can make patients fear and avoid the procedure, potentially making them fail to seek appropriate medical attention. A comprehensive investigation is warranted since some research focuses on the knowledge and awareness of tonsillectomy related to immune function. There are no add-ons or autoimmune diseases in Saudi Arabia, particularly in the Qassim province. This research will contribute to a better understanding of community knowledge and attitudes toward tonsillectomy in the region, potentially informing future public health initiatives and patient education efforts. Hence, this study aimed to address this gap by assessing adults’ awareness and perceptions in Qassim regarding the potential impact of tonsillectomy on the immune system, including its perceived relationship to autoimmune diseases.

## Materials and methods

This descriptive cross-sectional study targeted adults aged 18 and above in the Qassim Region to evaluate their knowledge and awareness regarding the effect tonsillectomy can have on a patient’s immunity and vulnerability to autoimmune diseases. The study was conducted over a six-month period, with the sample size being calculated using Epi Info software, version 7.2.2.6 (Centers for Disease Control and Prevention, Atlanta, Georgia, US). With a CI of 95%, a power of 80, and an expected frequency of 50%, the estimated sample size was 383. Respondents aged 18 and above and residents of the Central Region, Saudi Arabia, were recruited, while those less than 18 years and not Central Region residents were excluded. Data were collected using an online survey questionnaire distributed through social media. A convenience sample method was employed to recruit participants until we reached the required sample size.

Questionnaire

A self-administered online questionnaire was used to collect data in the Al-Qassim Region from August 18 to October 20, 2024. The questionnaire was created with Google Forms (Google, Mountain View, California, US) based on a previous study [10]. The questionnaire, available in both Arabic and English, consists of 5 sections and 17 close-ended questions. The questions cover various aspects, including demographic information, awareness of tonsillectomy and autoimmune diseases, medical history of tonsillectomy, and attitudes toward tonsillectomy and autoimmune diseases. Knowledge of the effect of tonsillectomy on immunity is assessed using a single question, "Do you think there is a relationship between tonsillectomy and autoimmune disease (the body attacks itself)?" Responses were classified into two categories such as "yes," considered as with knowledge, and "no/I don't know," considered as without knowledge.

Ethical considerations

Approval for the study was obtained from the Research Ethics Committee at the Qassim Health Cluster (No: H-04-Q-001) on August 18, 2024. Participants were informed about the study's purpose before participation. Since the questionnaire was distributed online, consent to participate was implied by completing the survey. All participants were voluntarily, and their responses were kept confidential and used solely for research purposes. The study declares no conflicts of interest or financial support from any organization and has no affiliations or involvement with any external entities.

Statistical analysis

Data analyses were conducted using SPSS version 26 (IBM Corp., Armonk, NY, US). Descriptive statistics described the sociodemographic features of the sample and the responses they provided. Univariate analyses were performed to determine the factors influencing the knowledge and awareness of the existence of an association between tonsillectomy and immunity level. A multivariate regression analysis was then conducted to establish the significant independent factor of the knowledge of the effect of tonsillectomy on immunity level, with corresponding odds ratios and 95% confidence intervals. Statistical significance was identified at p<0.05.

## Results

Table 1 presents the socio-demographic characteristics of the respondents. Of the 383 participants included in the study, a substantial proportion (263; 68.7%) of them were aged 18-30 years, with more than half 249 (65.0%) being females. Most (247; 64.5%) had a bachelor's degree or higher, and more than half (231; 60.3%) had a monthly income of less than 5000 SAR.

**Table 1:** Socio-demographic characteristics of the participants (N=383) Socio-demographic information presented in frequencies (n) and proportion (%)

Study Data	N (%)
Age group	
<18 years	20 (05.2%)
18 – 30 years	263 (68.7%)
31 – 40 years	69 (18.0%)
>40 years	31 (08.1%)
Gender	
Male	134 (35.0%)
Female	249 (65.0%)
Educational level	
Middle school	23 (06.0%)
High school	113 (29.5%)
Bachelor's or higher	247 (64.5%)
Occupation	
Unemployed	102 (26.6%)
Employed	131 (34.2%)
Student	150 (39.2%)
Monthly income (SAR)	
<5,000	231 (60.3%)
5,000 – 9,000	65 (17.0%)
>9,000	87 (22.7%)

Regarding awareness and perceptions of the impact of tonsillectomy on immunity and autoimmune diseases, more than half 209 (54.6%) of the participants believed that tonsillectomy can affect immunity. Nearly one-third (141; 36.8%) believed there is a relationship between tonsillectomy and autoimmune diseases while the majority 242 (63.2%) were unsure about the existence of this relationship. A significant majority of participants 233 (60.8%) believed that public knowledge about tonsillectomy was insufficient; nearly one-fifth (78; 20.4%) indicated that public knowledge was sufficient while only 72 (18.8%) were unaware of the topic (Table 2). 

**Table 2:** Assessment of awareness of the impact of tonsillectomy on the level of immunity and autoimmune diseases (N=383) Assessment of the participants awareness of the impact of tonsillectomy on immunity and autoimmune disease presented in frequencies (n) and proportion (%)

Awareness items	N (%)
Do you think tonsillectomy affects immunity?	
Yes	209 (54.6%)
No	106 (27.7%)
I don't know	68 (17.8%)
Do you think there is a relationship between tonsillectomy and autoimmune disease (the body attacks itself)?	
Yes	141 (36.8%)
No	127 (33.2%)
I don't know	115 (30.0%)
Do you think tonsillectomy can prevent certain diseases?	
Yes	223 (58.2%)
No	82 (21.4%)
I don't know	78 (20.4%)
Do you believe there are benefits to keeping the tonsils?	
Yes	231 (60.3%)
No	75 (19.6%)
I don't know	77 (20.1%)
Do you think public awareness about tonsillectomy is sufficient?	
Yes	78 (20.4%)
No	233 (60.8%)
I don't know	72 (18.8%)
Based on your knowledge of the relationship of tonsillectomy with immunity, have you decided to get operated on yourself or any of your relatives?	
Yes	180 (47.0%)
No	203 (53.0%)
Have you undergone a tonsillectomy?	
Yes	65 (17.0%)
No	318 (83.0%)
If yes, when did you have the surgery? ^(n=65)^	
<1 year ago	06 (09.2%)
1 - 3 years ago	05 (07.7%)
4 - 10 years ago	20 (30.8%)
>10 years ago	34 (52.3%)
If yes, did you experience any complications post-surgery? ^(n=65)^	
Yes	11 (16.9%)
No	54 (83.1%)
Have you or your family been advised by a doctor to undergo a tonsillectomy?	
Yes	178 (46.5%)
No	205 (53.5%)
Based on your knowledge, would you recommend tonsillectomy to others?	
Yes	207 (54.0%)
No	176 (46.0%)

It was observed that nearly half of the participants (180; 47.0%) expressed a willingness to undergo tonsillectomy themselves or have a relative undergo the procedure, based on their knowledge of the relationship between tonsillectomy and immunity. The prevalence of participants who had undergone tonsillectomy was 65 (17.0%). Among them, most (34; 52.3%) had the procedure more than 10 years ago. Of those, approximately 11 (16.9%) experienced postoperative complications. Furthermore, nearly half 178 (46.5%) had received advice from a doctor to undergo a tonsillectomy. More than half of the participants (207; 54%) would recommend the procedure to others.

**Figure 1:**
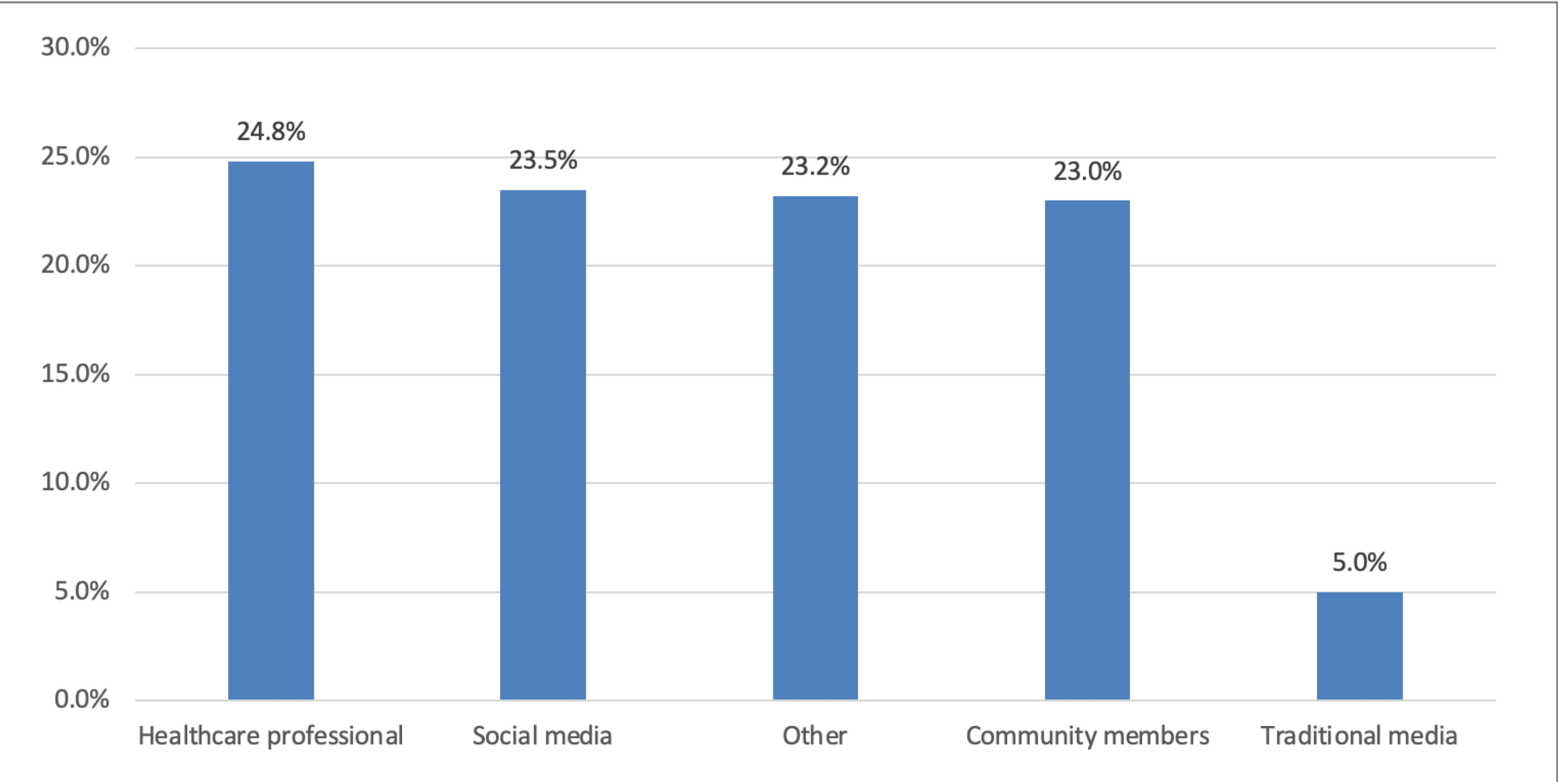
Source of information regarding tonsillectomy and immunity

Figure 1 illustrates that the most common source of tonsillectomy information was healthcare professionals (95; 24.8%), followed by social media (91; 23.5%) and community members (88; 23%).

In the univariate analysis, we observed that participants with a bachelor's degree or higher (p = 0.014), those who were employed (p = 0.033), and those with a monthly income of less than 5000 SAR (p = 0.030) were more likely to be aware of the impact of tonsillectomy on immunity. No significant relationships were found between awareness of the effect of tonsillectomy on immunity and age, gender, having undergone tonsillectomy, receiving advice to undergo tonsillectomy, or recommending tonsillectomy to others (p > 0.05) (Table 3).

**Table 3:** Univariate analysis of the factors influencing knowledge about the impact of tonsillectomy on the level of immunity (N=383) § P-value has been calculated using the chi-square test; ** Significant at the p<0.05 level

Factor	Knowledge of the impact of tonsillectomy on the level of immunity		P-value ^§^
	With knowledge N (%) (n=141)	W/O knowledge N (%) (n=242)	
Age group			
≤30 years	99 (70.2%)	184 (76.0%)	0.211
>30 years	42 (29.8%)	58 (24.0%)	
Gender			
Male	49 (34.8%)	85 (35.1%)	0.941
Female	92 (65.2%)	157 (64.9%)	
Educational level			
High school or below	39 (27.7%)	97 (40.1%)	0.014 **
Bachelor's or higher	102 (72.3%)	145 (59.9%)	
Occupation			
Unemployed	39 (27.7%)	63 (26.0%)	0.033 **
Employed	58 (41.1%)	73 (30.2%)	
Student	44 (31.2%)	106 (43.8%)	
Monthly income (SAR)			
<5,000	75 (53.2%)	156 (64.5%)	0.030 **
≥5000	66 (46.8%)	86 (35.5%)	
Have you undergone a tonsillectomy?			
Yes	26 (18.4%)	39 (16.1%)	0.559
No	115 (81.6%)	203 (83.9%)	
Have you or your family been advised by a doctor to undergo a tonsillectomy?			
Yes	71 (50.4%)	107 (44.2%)	0.245
No	70 (49.6%)	135 (55.8%)	
Based on your knowledge, would you recommend tonsillectomy to others?			
Yes	78 (55.3%)	129 (53.3%)	0.703
No	63 (44.7%)	113 (46.7%)	

In the multivariate regression model, it was found that participants with higher education were 1.63 times more likely to be aware of the impact of tonsillectomy on immunity compared to those with lower education (AOR = 1.632; 95% CI = 1.023-2.604; p = 0.040). No significant effects were observed regarding awareness of the impact of tonsillectomy on immunity in relation to occupation and monthly income after adjustments in the regression model (p > 0.05) (Table 4).

**Table 4:** Multivariate regression analysis to determine the significant independent predictor of knowledge about the link between tonsillectomy and autoimmune disease (n=383) AOR: adjusted odds ratio; CI: confidence interval ** Significant at the p<0.05 level

Factor	AOR	95% CI	P-value
Educational level			
High school or below	Ref		
Bachelor's or higher	1.632	1.023 – 2.604	0.040 **
Occupation			
Student	Ref		
Employed	1.523	0.885 – 2.620	0.128
Unemployed	0.960	0.528 – 1.744	0.892
Monthly income (SAR)			
<5,000	Ref		
≥5000	1.195	0.707 – 2.017	0.506

## Discussion

Tonsillectomy is a common procedure performed to prevent recurrent tonsillitis and improve patients' quality of life. Despite its widespread acceptance, misinformation about its impact on immunity and autoimmune function is growing, particularly in Saudi Arabia, highlighting the need for accurate information to dispel misconceptions surrounding the procedure [13,14]. This study aimed to address this gap by assessing the awareness of the adult population in the Qassim Region regarding these impacts and their potential link to autoimmune diseases.

In our study, nearly half of the participants (209; 54.6%) believed that tonsillectomy could affect immunity, whereas only 141 (36.8%) thought there was a relationship between the procedure and autoimmune diseases. Similarly, a study by Kwon et al. found that about 51.7% of participants were aware of the role of tonsils and related diseases, with approximately 47% indicating the need for more information about tonsillectomy [15]. Other recent studies, including those by Kim et al. and Jakimovski et al., suggest that tonsillectomy may increase the risk of vitiligo and multiple sclerosis, respectively [16,17]. However, a comprehensive review by Hu et al. reported no significant difference in immunity levels between pediatric patients who had tonsillectomy and those who did not, noting that long-term immune function did not decline following tonsil removal [18]. Additionally, the study by Plath et al. found that tonsillectomy in patients with recurrent tonsillitis resulted in a reduced incidence of upper respiratory tract infections and improved quality of life [19]. These discrepancies in study outcomes may be attributed to differences in methodology, sample sizes, and population diversity, highlighting the need for further studies using larger sample sizes.

The current study revealed inadequate awareness of tonsillectomy, with a notable proportion of participants 233 (60.8%) believing that public knowledge about the procedure was insufficient; about 72 (18.8%) were unaware of the topic. Only 78 (20.4%) participants indicated that public knowledge was sufficient. The study found that the most common sources of information about tonsillectomy were healthcare professionals (95, 24.8%), followed by social media 91 (23.5%) and community members 88 (23%). The study highlights the need to leverage social media as a crucial tool for community awareness, providing a platform for individuals to express themselves and gain valuable insights.

Despite inadequate awareness of tonsillectomy, there was a positive attitude toward the procedure. Nearly half of the participants (180; 47.0%) expressed a willingness to undergo tonsillectomy themselves or have a relative undergo the procedure, and more than half (207; 54%) were willing to recommend it to others.

The multivariate analysis identified education as a significant predictor of knowledge, with participants having higher education levels 1.6 times more likely to be knowledgeable about tonsillectomy. No significant association was found between participants' knowledge of the procedure's effects on immunity and whether they had undergone tonsillectomy (p = 0.559). Similar studies showed decreased IgA levels one month after tonsillectomy, but no significant changes after three months or recurrence of respiratory infections, suggesting no long-term impact on immunity [20,21]. In contrast, Radman et al. found lower IgM, IgA, and IgG levels in the tonsillectomy group [22]. Given the limited data on factors influencing knowledge about tonsillectomy's impact on immunity, further research is needed.

The findings of this study are subject to several limitations. First, the cross-sectional design can only assess relationships between study attributes, not their causalities. Second, there were notable variations in demographic factors, such as age, gender, and income, which may have made it difficult to generalize the pairwise comparisons of variables. Third, the use of an online survey for data collection may have introduced bias, as it was not possible to verify the accuracy of participants' responses.

## Conclusions

The study found inadequate awareness among the adult population in the Qassim Region of Saudi Arabia regarding the impact of tonsillectomy on immunity and autoimmune diseases. Public awareness programs and social media can play a crucial role in dispelling misconceptions about tonsillectomy and its effects on immunity and autoimmune diseases. There is a need for further educational interventions to challenge and correct public misconceptions about the relationship between tonsillectomy and immunity, as well as autoimmune diseases.
